# Smart TV-Smartphone Multiscreen Interactive Middleware for Public Displays

**DOI:** 10.1155/2015/534949

**Published:** 2015-04-09

**Authors:** Francisco Martinez-Pabon, Jaime Caicedo-Guerrero, Jhon Jairo Ibarra-Samboni, Gustavo Ramirez-Gonzalez, Davinia Hernández-Leo

**Affiliations:** ^1^Telematics Engineering Group, University of Cauca, Street 5 No. 4-70, 19002 Popayán, Colombia; ^2^Serra Húnter, ICT Department, Pompeu Fabra University, C/Roc Boronat 138, 08018 Barcelona, Spain

## Abstract

A new generation of public displays demands high interactive and multiscreen features to enrich people's experience in new pervasive environments. Traditionally, research on public display interaction has involved mobile devices as the main characters during the use of personal area network technologies such as Bluetooth or NFC. However, the emergent Smart TV model arises as an interesting alternative for the implementation of a new generation of public displays. This is due to its intrinsic connection capabilities with surrounding devices like smartphones or tablets. Nonetheless, the different approaches proposed by the most important vendors are still underdeveloped to support multiscreen and interaction capabilities for modern public displays, because most of them are intended for domestic environments. This research proposes multiscreen interactive middleware for public displays, which was developed from the principles of a loosely coupled interaction model, simplicity, stability, concurrency, low latency, and the usage of open standards and technologies. Moreover, a validation prototype is proposed in one of the most interesting public display scenarios: the advertising.

## 1. Introduction

Traditionally, public displays have played a role as static and nonpersonalized devices that broadcast information for many people indifferently. Nevertheless, modern approaches demand a new generation of public displays for pervasive environments, where they may be able to offer not only customized content but also interaction capabilities for users. Although several alternatives have been studied to make public displays smarter, one emerging model looks like the most interesting, the Smart TV initiative. Smart TV proposes a new generation of televisions and set top boxes with more processing power and a better Internet integration [1]. This emergent model also offers a valuable capability for connecting and sharing content with other devices like smartphones or tablets, via standards such as UPnP (Universal Plug and Play) or DLNA (Digital Living Network Alliance). This feature is extremely interesting for the new generation of public displays. In the past, several researches have developed interaction schemes between public displays and mobile devices using personal area network technologies like Bluetooth or NFC (Near Field Communication). This kind of solutions has been designed thinking in a particular domain or application and they do not consider a multiscreen cooperation scheme where the content may be distributed between the screens in a complementary way. Therefore, research about a new generation of public displays based on a Smart TV model is still incipient.

In spite of the efforts of several important vendors such as Samsung, LG, Google, or Apple, a real open platform for enabling multiscreen interactive applications is still nascent. For the purpose of this research, the multiscreen concept makes reference to the collaboration between the screens of different devices [2]. The Smart TV screen is supported by mobile devices screens that not only display complementary content but also enable interactions. Most of the approaches for Smart TV and mobile devices interaction are based on nonstandard techniques over HTTP (HypertText Transfer Protocol) like long polling, which looks to overcome the restrictions associated with the stateless condition of the HTTP protocol. This implementation demands more bandwidth and increases the protocol overhead that is problematic for public display environments. Additionally, the architectures are designed thinking on domestic environments that support applications with few clients at home. A Smart TV-Smartphone multiscreen interactive solution for public displays settings demands a scalable architecture and a robust message transport mechanism.

The proposed solution enables a multiscreen interactive middleware for public displays based on a Smart TV-Smartphone cooperation model. This approach includes a reference architecture to implement the middleware, and it defines an interaction model based on the publish/subscribe paradigm. Middleware features are analyzed from important requirements for public display environments such as scalability, low latency, simplicity, concurrency, the use of open technologies, and a loosely coupled interaction model. We provided a reference implementation for the Android platform and it was validated in the advertising field, specifically in digital signage environments where public displays are required.

The paper is structured as follows.  Section 2 summarizes some related works in the last years.  Section 3 presents an architectural approach for the middleware. The next section summarizes some aspects related with the middleware communication model.  Section 5 describes the experimental framework for validation and evaluation purposes.  Section 6 discusses the experimentation results. The last section introduces some conclusions.

## 2. State of Art

The new generation of public displays has inspired several research works with different perspectives in the last years. However, they share a strong common motivation: a better experience from the user interaction perspective [3]. Specifically, this scholar focuses on the interaction with public displays using personal devices like smartphones or tablets. This section introduces some researches related to the interaction mechanisms for public displays, highlighting mayor interaction requirements for the Smart TV model. Then, we describe some existing middleware and toolkits for public displays and finally we analyze some toolkits and Smart TV approaches.

### 2.1. Public Displays Interaction Mechanisms

For years, several approaches have been studied about content adaptation and interaction schemes between public displays and smartphones. For example, [4] analyzes the interaction throughout gestures during a screen replication and [5] introduces a touch screen interaction supported on NFC capabilities. However, both approaches do not consider a real collaborative interaction between the devices and a screen replication approach is preferred instead. Other researches consider some interaction models for including zoom functions for the main public display content in the mobile devices [6, 7], as long as others consider some phone sensor functionalities [8, 9]. Nonetheless, none of the previous approaches proposes a real collaborative multiscreen scheme between public displays and mobile devices; these solutions are addressed to specific applications or functions, which do not include a more generic middleware platform support that may be applied to several domains.

Although the mobile devices provide an obvious mechanism through which viewers can express their preferences and communicate them to the nearby public displays, the possible interaction mechanisms may be diverse. According to [10] these interaction techniques can be grouped into two major approaches.

The first group uses a custom application designed for the specificities of a particular displays system in a well-defined scenario; this dependence makes these applications useless for any other domain. Some researches of the University of Oulu in Finland [11], Lancaster University in England [12], University of Stuttgart in Germany [13], and University of Ottawa in Canada [14] are some examples of this first group. As an important contribution, these authors define several design requirements for a public display system deployment, but these experiments neither consider the use of Smart TV as an Interactive Public Display nor interaction schemes throughout smartphones that enable multiscreen features.

On the other hand, the second group approaches depend on the availability of several communication alternatives in the mobile devices such as Bluetooth, NFC, or IVR (Interactive Voice Response). Research works presented in [15–19] or [20] are examples for this second group. This form of interaction is very attractive to support an interaction almost universal. However, [10] remarks that these approaches “*may be limited in their ability to frame the interaction with regard to personalization and the shared meaning of the interaction*.” In this sense, a Smart TV-Smartphone cooperation scheme may be an interesting research alternative to implement robust multiscreen interaction schemes.

### 2.2. Public Displays Middleware and Toolkits

There are some related works on middleware systems which provide different levels of abstraction for communication complexities and different interaction modes for public displays like MAGIC Broker [21], OSGi Broker [22], or UbiBroker [23].

The MAGIC Broker provides a set of common abstractions and a RESTful Web services based protocol to develop interactive public large screen display applications using mobile device interactions. For this purpose, the RESTBroker uses a set of common abstractions such as channels, events, states, services, and content; specifically, the channels describe entities in the environment and they support a hierarchical structure. The events and state information are routed based on the channels, something similar to a topic in the publish-subscribe pattern implemented over HTTP. Otherwise, OSGi Broker is an evolution of MAGIC Broker, which replaces the RESTBroker to add support for SMS (Short Message Service) and VoiceXML (Voice eXtensible Markup Language) inputs. Both RESTBroker and OSGiBroker use the notion of channels following a publish-subscribe design pattern to decouple message sources from message sinks.

On the other hand, the UbiBroker is an event-based communication architecture for a pervasive display network. It is based on a proven and widely used general purpose open source publish-subscribe messaging software; also, it addresses several major requirements for pervasive display networks including decoupling, stability, simplicity, and interoperability. Regarding mobile devices interaction, UbiBroker supports several devices throughout hotspots user interfaces implemented using a Web paradigm. However, a real implementation for the Smart TV model is not obvious.

According to the previous analysis, it is important to remark that the current research looks for enabling more attractive ways of interaction with public displays, using the gestures commonly implemented in the mobile operating systems. According to our experience, a native application implementation supports more fluent and stable interactions in a real public display scenario than a Web implementation as UbiBroker suggests.

### 2.3. Smart TV Toolkits and Platforms

Smart TV model exposes interesting features to extend the interaction capabilities of conventional public displays, thanks to its ability to connect other devices like smartphones or tablets. Platforms like Samsung Convergence Framework [24] or Samsung MultiScreen SDK [25] have been the first steps to build Smartphone-Smart TV interaction schemes. The first one defines a proprietary model for the connection of Smart TV and smartphones based on RESTfull endpoints in a HTTP long polling communication model under a Local Area Network environment; the second one defines a proprietary SDK for the connection of Smart TV and Smartphone based on cloud services using WebSockets communication model [26]. Our previous work deploying a Smart TV-Smartphone cooperation model in digital signage reveals that Samsung Smart TV Convergence Framework has some restrictions related to the maximum number of concurrent connected users and some intermittent behavior during paring and connection process [27]. Moreover, the use of proprietary platforms restricts the adaptability of the system.

Other alternatives are related with box TV platforms like Google TV and Apple TV; the first one provides the Android platform ecosystem support for the development of third party applications, but the second one is still closed to third party developers.

A deeper analysis of the previous approaches reveals a common issue between them. Most of the interaction has been designed thinking in the mobile device as an input device and the Smart TV as an output device, which restricts the operation of a real multiscreen cooperation scheme. On the other hand, the use of techniques as HTTP long polling in the communication strategy imposes restrictions in the stability and scalability of a system supporting multiple users on public displays interaction environments.

This research proposes an open middleware to support multiscreen interaction schemes based on the Smart TV-Smartphone model capabilities. It looks to overcome the current restrictions in order to support the public displays environments requirements.

## 3. Middleware Architecture

In this section, we will describe the proposed architecture for the middleware. At first, we introduce some specific requirements for a middleware supporting public display environments; they were defined according to our experience during the research and the contribution of previous related works.

### 3.1. Requirements

#### 3.1.1. Loosely Coupled Communication Model

In a typical public display-mobile device interactive system, users trigger actions by sending commands through their smartphones to get an answer from the public display. It demands a highly dynamic environment with support for ad hoc communications between the related nodes, to avoid complex setup hard-coupled interfaces. According to previous works [21, 23, 28, 29], an event-based communication model supporting a publish/subscribe interaction scheme may be the best alternative to meet these requirements.

#### 3.1.2. Stability

The middleware has to provide a reasonable availability and a small number of crashes. Moreover, [23] remarks “It is challenging to achieve stability if communication middleware is built from scratch, as reliability tends to come over time after careful testing and sustained development support,” so it is advised to use proven and well tested communication solutions as starting point.

#### 3.1.3. Open

One of the most important considerations during the middleware development was the ability to evolve supporting standard and open technologies. The use of standard open protocols, open source implementations, and open development platforms also eases the integration of third party applications.

#### 3.1.4. Simplicity

The middleware should be designed thinking about third party developers, so the simplicity is a key requirement for them. Simplicity involves a well-defined API using software design patterns that enable its use and extension by third party apps.

#### 3.1.5. Concurrent Interaction

Middleware should support multiple concurrent interactions from different users in front of the same public display. The number of supported users should be limited by the public screen size.

#### 3.1.6. Low Latency Communication

The current mobile devices have a fluent and fast answer to user interactions most of the time. It is important to keep these features as long as the user is interacting with the public display from his smartphone/tablet in order to improve the user experience.

### 3.2. Architecture

**Figure 1:**
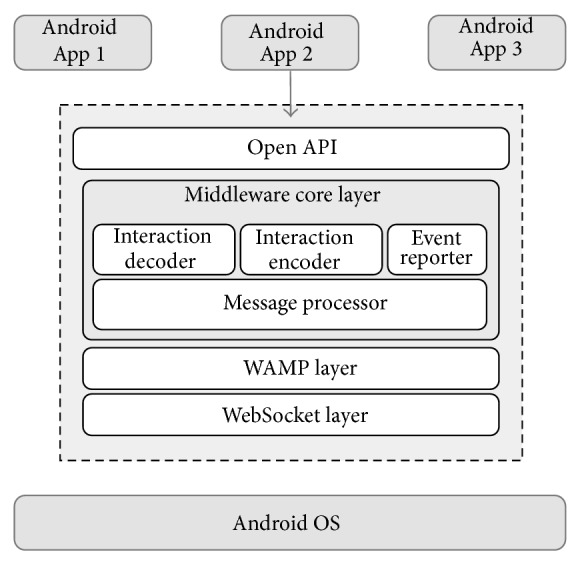
Middleware architecture.

Figure 1 shows the reference architecture for the middleware. The proposed architecture has a layered structure that decouples its main components functions; it also eases the middleware maintenance and its evolution over the time. At the bottom, this layered architecture is supported by the Android OS capabilities, mainly by a rich set of networking features; the upper layer offers an Open API that allows the access to the middleware functions by third party apps in a simple manner, abstracting the underlying complex functions. Next, a brief description of each layer will be introduced starting at the bottom layer.

#### 3.2.1. WebSocket Layer

A lightweight WebSocket protocol version was implemented to get a full real-time bidirectional communication between mobile devices and public displays [26]. Currently, several attempts to provide real-time communication using Internet Protocols rely on nonstandard techniques over a stateless protocol like HTTP; these techniques are known as Comet [30]. Polling, long polling, or streaming are some of the most popular HTTP Comet mechanisms, which postpone the HTTP response until the server has something to send to the client, simulating a real-time communication [31]. Nonetheless, the Comet techniques increase the latency and the protocol overhead, which restrict their application in the development of a middleware for user interaction purposes. The WebSocket protocol, instead, provides a full-duplex, bidirectional communication channel that operates through a single open socket over the Web, a desirable feature to build scalable real-time applications [32]. By reducing the overhead, the WebSocket Layer provides an efficient bidirectional communication channel between different devices with a low latency, one of the most important requirements for real-time user interaction systems [30, 31].

#### 3.2.2. WAMP Layer

The proposed middleware can be defined as a message-oriented middleware (MOM) using a publish/subscribe interaction paradigm. MOM is a specific class of middleware that supports the exchange of general-purpose messages in a distributed application environment. The most sophisticated MOM systems ensure message delivery by using reliable queues and providing directory, security, and administrative services required to support messaging. Publish/subscribe is a popular interaction paradigm where the sender is called the publisher and the receiver is called the subscriber. One publisher can publish a message to many consumers through a virtual channel called a topic. Consumers choose a topic of their interest to subscribe to it; any message for a specific topic is delivered to all the subscribers [33].

According to the previous description, this layer handles all the complexities related to the message interchange using a publish/subscribe interaction scheme. An open source Android implementation for Web Application Message Protocol (WAMP) was modified to assure stability and availability [34]. The high level WAMP protocol is supported by the WebSocket protocol, which makes the information exchange between publisher and subscribers truly asynchronous. Moreover, the notifications of events for subscribers are more efficient thanks to the overhead reduction. Therefore, the WAMP Layer provides a good efficiency/speed relationship, an important requirement to send information at proper times when the interaction between mobile devices and Smart TV public displays takes place.

#### 3.2.3. Middleware Core Layer

The integration of several technologies and software design patterns may help to get the middleware expected behavior. Middleware Core Layer acts like a gateway for the messages exchange and it manages the interaction process according to a well-defined communication model. Users touch screen actions are preprocessed and then prepared as formatted messages that are sent to the other devices. Likewise, incoming messages are processed and delivered to third party Smart TV applications throughout the Open API.

The Middleware Core Layer functions rely on particular and more specialized components as follows.

- *Interaction Encoder*. All touch events on the Smartphone screen need to be properly encoded and prepared prior to publishing them on the public display. Mobile applications sense the device screen and detect touch events and gestures, which triggers system touch events such as press, long press, tap, double tap, swipe, drag, move, or even a custom touch event.
- *Message Processor*. This component is responsible for sending and receiving messages to and from other devices throughout the WAMP Broker. Encoded touch events can be sent to the WAMP Broker by publishing messages with encoded information about a specific topic. The WAMP Broker defines the final destination for those messages throughout different notifications to the subscribers of that topic. Then, it reads the incoming messages and delivers the content to the interaction decoder.
- *Interaction Decoder*. All incoming encoded information needs to be properly decoded in order to rebuild the original touch event. These rebuilt touch events are sent to the mobile application using the Open API callback methods.
- *Event Reporter*. A middleware activity log is possible thanks to the storage of execution and interaction data, collected throughout Flurry Analytics API [35]. Execution data have information about how often an application was used and it may be useful for crash reporting purposes to third party developers. On the other hand, interaction data deliver information about how many users interacted with the system and the way they did it. This information may be an indicator to measure the success of an application or advertising campaign, for example.

#### 3.2.4. Open API

It is the main entry point for third party mobile/public display applications; the Open API abstracts the middleware capabilities and low-level communications protocol handling to the developers. A set of development patterns were implemented, so the application developer can use* factories* for clients and message creation,* observers* for connection, events, control and feedback notifications, and* singletons* for user interaction detection [36]. During the API development process the support for additional UI components other than the Android widget toolkit was not considered; the API development process was focused on the abstraction of the communication complexities. It keeps the API light and simple, one of the requirements described at the beginning of this section. The next section includes a more detailed description about the Open API functions.

## 4. Communication Model

**Figure 2:**
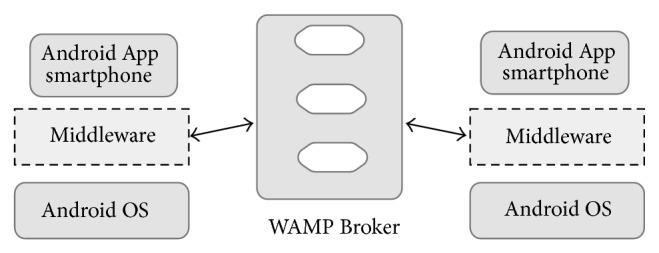
Pub/Sub communication model.

Message-oriented middleware is increasingly adopted as an enabling technology for modern event-driven applications [37]. Publish-subscribe paradigm is frequently used on well-known software architectures and technology domains such as Enterprise Service Bus (ESB), Enterprise Application Integration (EAI), Service-Oriented Architecture (SOA), and Event-Driven Architecture (EDA) [38]. The proposed middleware architecture uses this approach to connect mobile devices and smart public displays in a scalable way (Figure 2).

The publish/subscribe paradigm, as used in MOM systems, enables the devices to subscribe to topics of interest. Publish/subscribe MOM relies on asynchronous communications and it enables data-centric communications; according to this model, the consumers subscribe to the information they are interested in, and the publishers make these data available [39]. Typically, the publisher publishes messages to a broker on well-known topics, and the subscriber subscribes to these topics. On the other hand, the broker also assures the message delivery, so a message is not erased until all subscribers to a specific topic have received the data from the broker; it makes the communication model lighter for the clients. This is an important requirement for a Smart TV-Smartphone multiscreen middleware for public display purposes, because simultaneous end-to-end connectivity with all subscribers is not always guaranteed.

### 4.1. Interaction Model

**Figure 3:**
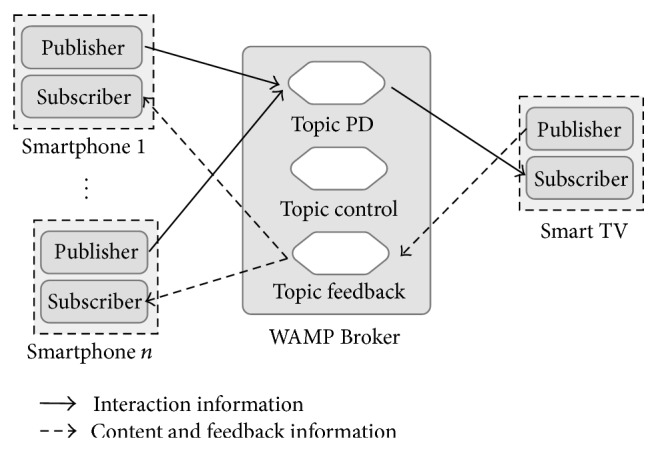
Interaction, content, and feedback information exchange.

In a Smartphone-Smart TV environment for public displays, one of the most important tasks is the ability to send information about the user touch screen interaction in the mobile device to the public display. So, the mobile device must publish appropriate messages on a specific topic in the WAMP Broker. At the same time, the Smart TV must subscribe to that topic to receive messages from all connected clients (Figure 3).

**Figure 4:**
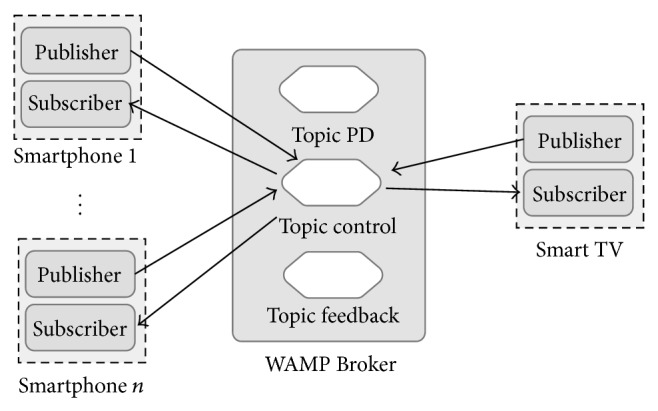
Control information exchange.

In this kind of environments, the Smart public display needs to know more details about the connected mobile devices. For example: How many of them are connected? When a new Smartphone is ready to publish messages for a specific Smart TV topic? Or when a Smartphone disconnection has taken place? The proposed interaction model provides a mechanism to handle these issues using a special topic where Smartphone and Smart TV can share special control messages (Figure 4). Throughout this special topic, application developers can implement sophisticated controls structures when required.

Otherwise, there are also some scenarios where Smart TV needs to share content and information to a particular device or group of devices. As in the previous case, the middleware uses a special topic where all devices can exchange supplementary information not related with interaction or control messages (Figure 3). For example, this special topic may transport the movie short description in a public display showing a movie list, improving the user comprehension of the content [40].

### 4.2. Protocol Description

Proposed middleware uses a well-defined protocol to enable the message interchange according to publish/subscribe paradigm.  Table 1 shows the structure for a generic frame and Table 2 shows the structure for message transporting touch events information.

Complex touch screen gestures like a swipe or drag over the screen are composed by several motion events, which are encapsulated throughout MotionEvent objects; however, only two of these objects are required to store the whole information. To publish a message with a MotionEvent object, it must be serialized and formatted using JSON format; with this structure, serialized data can be encapsulated into the message using the startContent, endContent, or Extras fields. Furthermore, convenience messages were defined for the most common user interaction actions. Being Android, the ground platform for the middleware reference implementation, there is a correspondence between Android touch gestures and convenience messages. As an example, Table 3 shows some convenience messages for Tap and Swipe gestures; however, a full set of messages support other gestures like Move, Press, Long Press, and Double Tap.

### 4.3. API Description

A small set of classes and interfaces were designed to make the communication management easier for third party developers.

**Figure 5:**
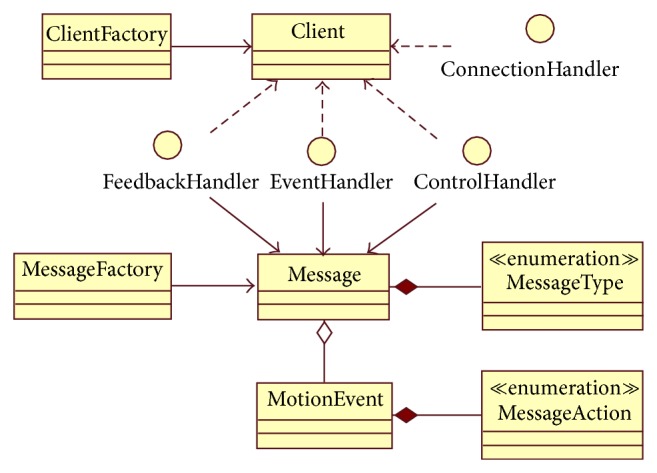
API class diagram.

Figure 5 shows a class diagram for the API. Next, a brief description of the API main classes will be presented.

- *Client*. Middleware main class to set up a connection with WAMP Broker: it also enables the publishing and subscribing process to different topics. Furthermore, this class supports the information pickup for log purposes.
- *ClientFactory*. Factory pattern implementation for *Client* objects creation, according to specific Handlers and parameters.
- *ConnectionHandler*. This interface defines callback methods for handling notifications about connection events to the WAMP Broker.
- *Event Handler*. This interface defines callback methods for handling notifications about the user interaction over the touch screen.
- *ControlHandler*. This interface defines callback methods for handling notifications about control events from publishers and subscribers.
- *FeedbackHandler*. This interface defines callback methods for handling notifications about events published in the feedback topic.
- *Message*. This class encapsulates the content structure for the whole messages interchanged between the clients (publishers/subscribers) and the WAMP Broker. Each message has a type, which is defined in the Enumeration MessageType, a source id, a payload that may be a serialized MotionEvent object and extra data.
- *MessageFactory*. Factory pattern implementation for* Message* objects creation, according to specific parameters: it defines convenience methods for objects creation according to the type of touch event generated from the user interaction.

According to the previous description, the setup for message interchange between smartphones and Smart TV using the API is so simple: a peer only needs to get a client instance from the ClientFactory and then publish or subscribe to the desired topic.

## 5. Experimental Framework

### 5.1. Motivation Stage and Prototype Description

One of the most interesting scenarios for public displays is related with advertising. Although several researches have focused on better mechanisms for ads delivery on personal devices like mobile phones, the ads delivery in public spaces, including malls or streets, is so attractive. This trend, known as digital signage is still very important for advertisers taking into account that the 75% of the purchase decisions take place in the shopping places or close to them [41]; specifically, these are the preferred places to locate public displays. On the other hand, the advertising campaigns may evolve from mainly static to more engaging experiences, if the users interaction with the public displays is improved beyond an attractive looking and feel to watch the ads in the screen; it is something valuable for advertisers with persuasion purposes. In this sense, the proposed middleware is a great support for these high interaction environments.

**Figure 6:**
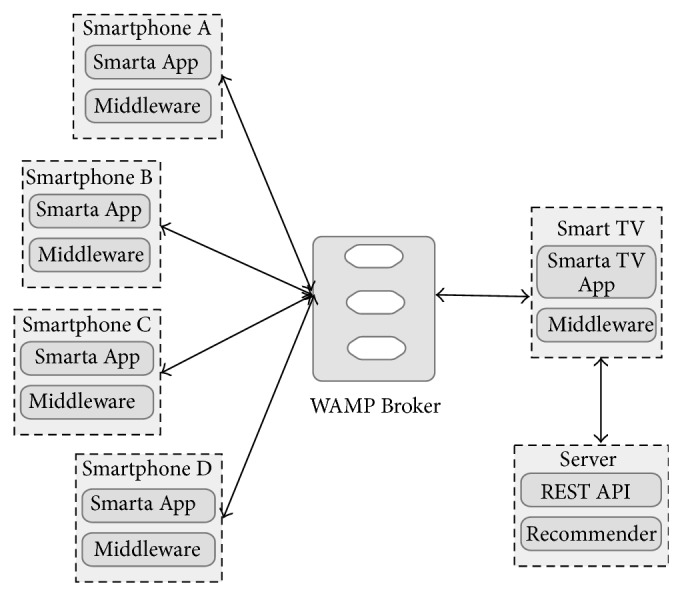
Prototype architecture.

With this motivation in mind, a little prototype for experimentation purposes was developed and tested in an academic environment. The prototype basis was to implement an electronic alternative to a traditional static ads board, in which people post ads using paper posters; these boards are frequently found on small shops or academic campus. The proposed implementation replaces the old board by a new cooperative Smart TV-Smartphone model, where both devices screens are offering ads to users under different but complementary approaches: ads recommendations for group profiles on public TV screen and ads recommendations for individual profiles on the smartphones screens. Moreover, the interaction capabilities between both devices change the static behavior of the traditional board; the basic architecture for the prototype is shown in Figure 6.

In summary, Android applications were developed for mobile phones and Google Smart TV set top box. These applications used the middleware API for interaction management and the users in front of the public display login to the system using a “Login with Facebook feature.”

Briefly, some of the main functionalities of the implemented prototype were as follows.

**Figure 7:**
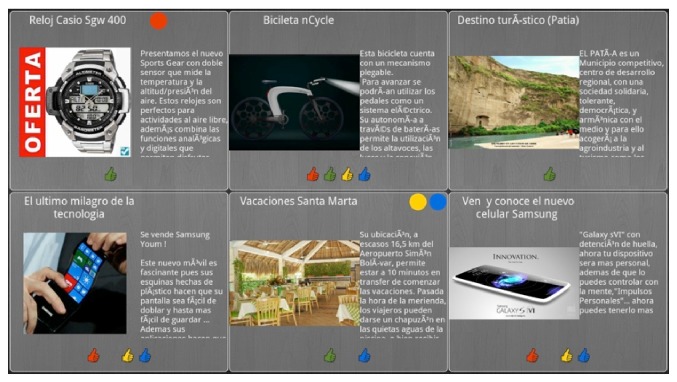
Public display ads screen.

*(i) Ads Recommendations for a Group of Users Watching the TV Screen*.  The groups of users were limited to four people, basically for usability reasons taking into account the screen size (42 inches). The recommender calculates the best ads list for the people interacting with the public screen; these ads were organized on list of six ads each (see Figure 7); the recommender implementation is out of the scope of this paper.

**Figure 8:**
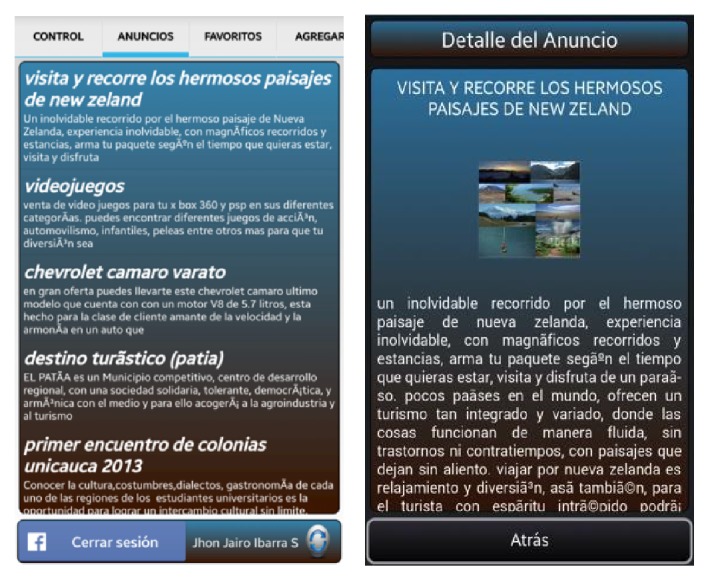
Mobile device adds list screen.

*(ii) Ads Recommendations according to Individual Preferences on the Smartphone Screen*.  The recommender calculates the best ads list for each person in front of the public display and it shows them on the mobile device screen (see Figure 8). The user can get detailed information for a particular advertisement and add ads to Favorites.

**Figure 9:**
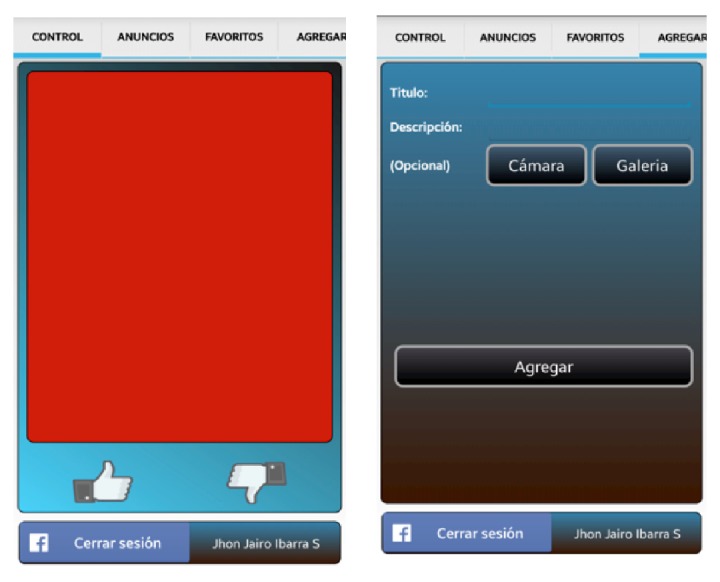
User pad control and ads post.

*(iii) Basic Interaction between Mobile Device Application and Public Display*.  The user can go over the ads on the public display using a control pad from the mobile application (Figure 9); using a tap gesture the user can get detailed information for a particular ad in public display and watch it on the mobile device screen. Each user is identified by a specific color (Figure 7).

*(iv) Rating Ads*.  User can rate the ads on public screen or mobile device list using “Like” and “No Like” options available in the mobile application user interface. For public display, each user is identified by a specific color (Figure 7).

*(v) Posting Ads*.  Users post ads to the public screen, writing the ad information from their mobile device. The user can use the smartphone camera or its photo gallery to upload a product picture (Figure 9).

*(vi) Interaction Logs*.  Thanks to the middleware capabilities, both applications are enabled to store interaction activity logs for each user action. For example, in this scenario the logs information was useful to generate implicit ratings according to the user action (request more info about an ad, ignore ads from the screen, add the ad to favorites, etc.).

### 5.2. Evaluation Methodology

Frequently, performance related aspects are the most common evaluation parameters for a message-oriented middleware (MOM). So, the system should be tested in almost-real scenarios and also in extreme conditions [42, 43] to find out reliable information about its performance. According to the previous arguments, online and offline tests were performed in two phases to observe the system behavior.

#### 5.2.1. Offline Experimentation

**Figure 10:**
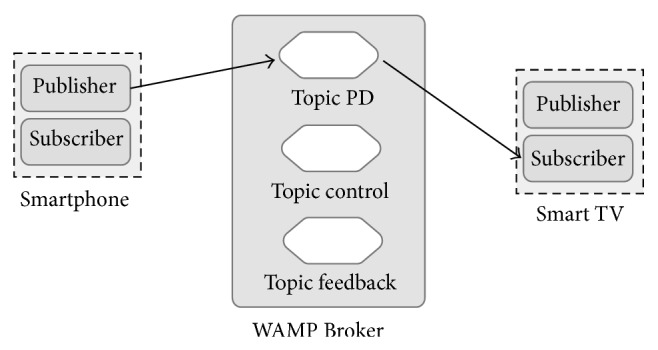
Offline test architecture.

During this phase, simulated components generated high traffic conditions to the middleware.  Figure 10 shows the basic architecture for the testing scenario. During these tests, different hardware devices sent and received messages at different rates with different computational load.  Table 4 summarizes the hardware features setup for this phase.

#### 5.2.2. Online Experimentation

During this phase, real users participated in the advertising scenario described in the previous section. As long as the offline tests are focused on the system performance under extreme conditions, the online tests try to simulate a real public display scenario for the middleware operation. As the middleware interaction protocol is based on gestures information sent from mobile devices, these tests enable the evaluation of the middleware performance when real users send real gestures using the API from the Android applications; this feature was not evaluated during the offline tests. For this purpose, twenty-four (24) students, eight (8) women and sixteen (16) men from the Electronics and Telecommunications Engineering program from the University of Cauca, with ages between 20 and 25 years participated in the experiment. Groups of 4 people were set up randomly to interact with the system during at least five (5) minutes periods.

The specific tests description and the results will be described in the next section.

## 6. Discussion

Next, we will analyze the most relevant results according to the evaluation methodology proposed in the previous section.

### 6.1. Offline Tests

#### 6.1.1. Test Number 1 Maximum Sustainable Throughput

**Figure 11:**
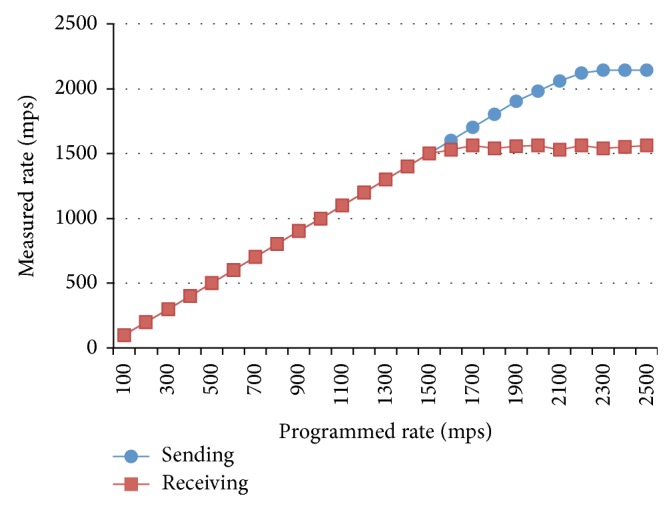
Sender and receiver rates comparison.

The purpose of the first test is to find the Maximum Sustainable Throughput (MST). A single subscriber and a single publisher were configured during the test. The publisher was sending messages with 64-byte length at a rate below 1500 messages per second (mps). Moreover, the receiver rate was almost the same as the publisher-sending rate. When the publisher sending rate was increased beyond 1500 mps, the receiver keeps the receiving rate around the 1500 mps; the sender increased the sending rate up to 2000 mps.  Figure 11 summarizes the system behavior at different sending rates. According to the graph, the MST for the middleware is 1500 mps (with hardware support features described on Table 4).

#### 6.1.2. Test Number 2 Effective Rate at MST

**Figure 12:**
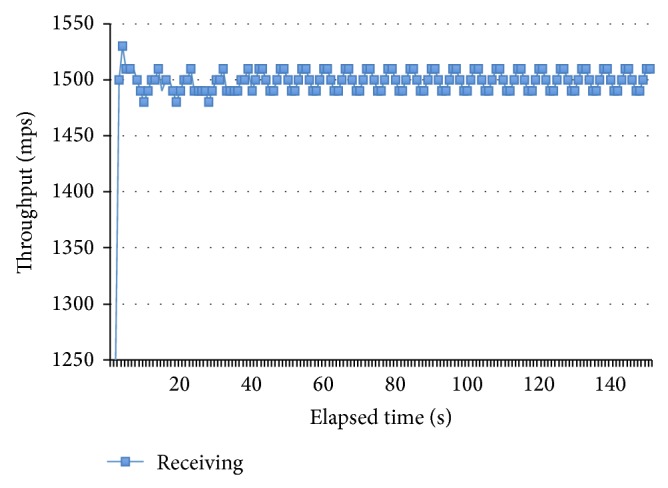
Subscriber receiving rate at MST.

Once the MST has been calculated, the second test was executed in similar conditions to the first test but this time the publisher-sending rate remained constant in 1500 mps to observe the subscriber receiving rate at different points in the time. The results in Figure 12 show the system stability to the MST throughput.

#### 6.1.3. Test Number 3 CPU Consumption at MST

**Figure 13:**
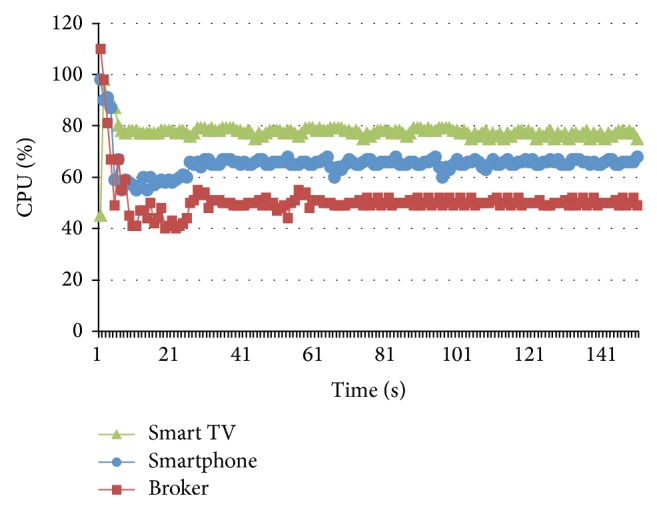
CPU consumption at MST.

Some measurements about CPU consumption were performed over the hardware components (Smart TV, Smartphone, and Broker) in different points over the time working at MST (1500 mps).  Figure 13 shows a CPU high consumption in all devices during the first five (5) seconds of the test; it is due to the connection process and the setup of the networking threads. However, ten (10) seconds later the behavior starts to be stable. On the other hand, it is possible to observe a higher CPU consumption for the Smart TV; it is due to a faster microprocessor available in the Smartphone.

#### 6.1.4. Test Number 4 MST with a Different Number of Publishers

**Figure 14:**
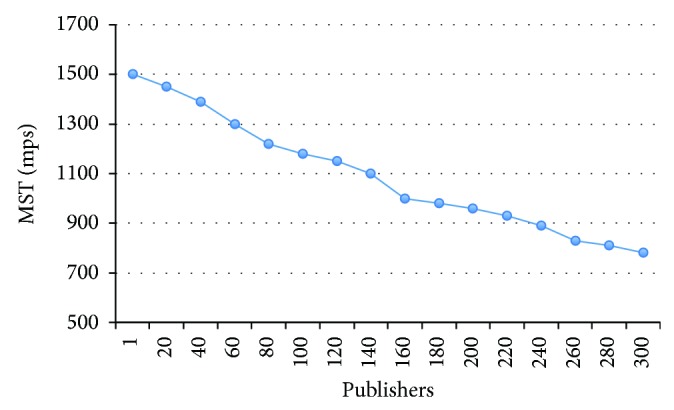
MST versus number of publishers.

This test observes the behavior of the MST when the number of publishers increases. So, one subscriber and 64 bytes length messages were configured during the test. The results are summarized on Figure 14. As expected, the number of publishers affects the MST adversely; with one publisher, the MST keeps the 1500 mps rate as in Test number 1, but it descends to 780 mps when 300 publishers are sending messages at a 10 mps rate. Anyway, it may be an extreme rate for most of the real public display scenarios.

#### 6.1.5. Test Number 5 MST with a Different Number of Subscribers

**Figure 15:**
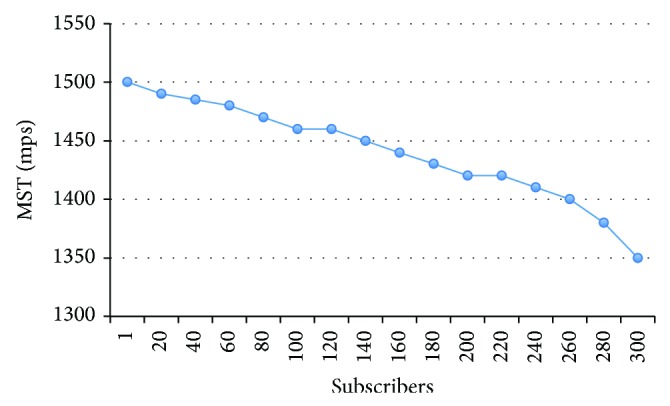
MST versus number of subscribers.

This test observes the behavior of the MST when the number of subscribers increases. One publisher and 64 bytes length messages were configured during the test. The results are summarized on Figure 15. Like the previous test, the number of publishers affects the MST adversely; in this case, the MST descends to 1350 mps when 300 subscribers send messages at a 10 mps rate. However, the impact is lower because most of the work is performed in the broker.

#### 6.1.6. Test Number 6 MST with Different Message Lengths

**Figure 16:**
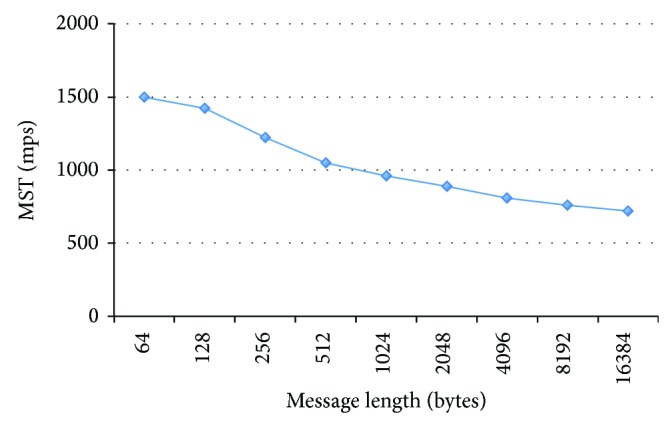
MST versus message length.

This test observes the behavior of the MST when the message length changes. One publisher and one subscriber were involved, changing the message length and calculating the MST for each case. In Figure 16 it is possible to observe how the MST decreases as long as the message length increases, because longer messages require more bandwidth and also more computational resources.

#### 6.1.7. Test Number 7 Connection Time and Latency

This test evaluates the connection times and the latency. The first one is defined as the time between the WebSocket open request from the client and the WAMP Broker answer. Otherwise, the latency measures the time between a message posted by the publisher and the message reception by the subscriber. One subscriber and one publisher were configured sending 64 bytes length messages. The results over 1000 samples are summarized in Table 5.

### 6.2. Online Tests

**Figure 17:**
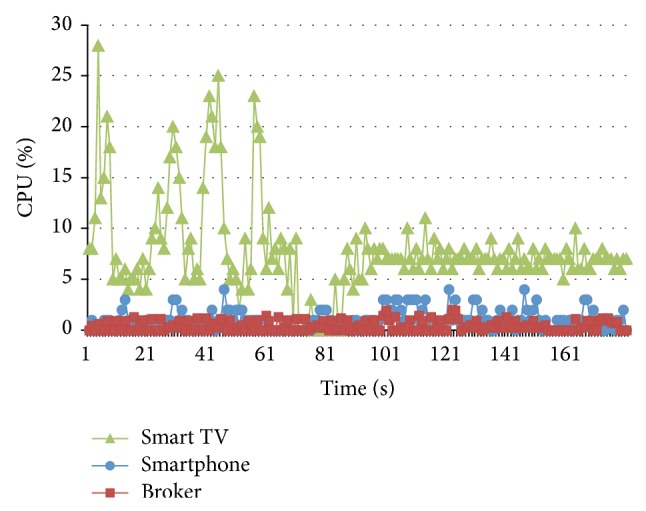
Online test CPU consumption.

**Figure 18:**
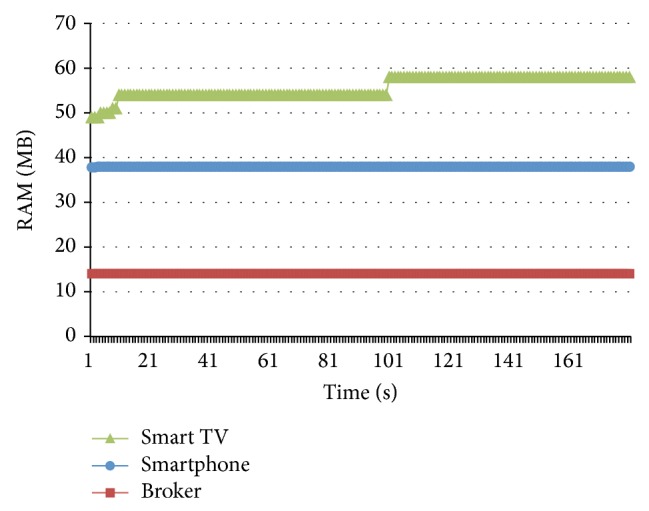
Online test RAM consumption.

According to the previous description, the offline tests were focused on the overall system performance under extreme conditions. The online tests were applied in a real and common scenario in a public display environment, with four (4) users in front of the screen interacting with the electronic ads board prototype described in Section 5. The number of users was restricted according to the screen size (42 inches) to assure a better experience for the volunteers during the experiment. Therefore, four publishers (4 smartphones) and one subscriber (the Smart TV) were used during the test to measure the CPU and RAM consumption for each component during five (5) minutes observation periods, according to the methodology described in the previous section. Figures 17 and 18 show a low CPU/RAM consumption and a rather stable behavior; it is interesting to observe the four high peaks, which take place when each user is requesting a new connection and a new set of group recommendations is calculated. In summary, the online tests show a good middleware components performance with low computational resources consumption, suggesting a good behavior on most of the real public display scenarios.

## 7. Conclusion

This research proposes an interactive Smart TV-Smartphone multiscreen middleware for public displays. Previous implementations for enabling public displays-smartphones interaction frequently were domain or technology specific. On the other hand, previous efforts by some vendors about the rising Smart TV model have implemented solutions based on HTTP techniques like long polling, which compromises the stability and the scalability of an interactive middleware for public display purposes. The contributions of the proposed middleware may be analyzed from the requirements defined previously at the beginning of Section 3.

Based on previous findings, we design and implement a scalable message-oriented middleware following a publish/subscribe paradigm to get a* loosely coupled communication model*. Furthermore, the proposed middleware layered architecture eases the decoupling between the complexities related with the low-level communication protocols, the interaction model for messages interchange, and the business logic of each third party application for a specific domain. In the same way, we design an* Open* protocol based on standard technologies like WebSockets and JSON using open source implementations to support the Smart TV-Smartphone cooperation scheme.

The design of an open architecture for the middleware based on the publish/subscribe paradigm improves the* stability* of the connections over other alternatives based on nonstandard HTTP techniques like long polling, an important requirement for public displays environments where multiple users must be supported. The proposed middleware had a stable behavior during all online and offline tests, compared with our previous work using the Samsung Convergence Framework [24, 27].

Otherwise, we design a* Simple* API over a widely supported platform like Android, which enables the use of most of the capabilities of the current mobile devices. Simple extensions would enable the use of hardware features (GPS, accelerometer, gyroscope, etc.) to motivate the development of creative and engaging applications. For example, accelerometer events may be used to simulate how objects are thrown to the public display; other modes may be created to engage the users in a competition using strength or speed gestures from the mobile phones. Therefore, this reference implementation enables the use of most of the set top boxes based on the Google platform.

About the* Concurrent Interaction*, the middleware uses a simple design based on gestures and messages interchange between the Smart TV and the mobile devices in a public display environment, which improves the scalability of the system. Although the prototype showed a good performance for four concurrent users, the offline tests evidence a good concurrent connections behavior still in extreme conditions.

Moreover, the middleware showed a* Low Latency* for the pool of connections during online and offline tests; it suggests the middleware applicability in several scenarios related to advertising, including home environments. A lot of possibilities are open to design creative multiscreen applications for entertainment purposes, including interactive multiscreen games thanks to the almost real-time transport message support provided by the middleware. Other scenarios like E-learning may be also interesting; for example, multimedia sessions oriented by the teacher on a Smart TV may be followed with complementary content in students' mobile devices at the same time. We keep in mind that all of these possibilities can be effectively addressed with low latency features.

About the middleware application, although the Android platform was selected to build a reference implementation for the middleware, the proposed architecture may be replied to other platforms; a new generation of open Smart TV boxes could be built over platforms like Raspberry Pi; for example, something that may be attractive for emergent markets.

Regarding the middleware evaluation, the offline tests show a promising performance on extreme conditions with average hardware capabilities, as long as the online tests with real users show a very good performance in a nonintensive public display scenario for the advertising domain. More demanding public display scenarios may be implemented using larger screens with Smart TV boxes support, using better hardware resources; as a matter of fact, the architecture was designed to be extensible from domestic places to more demanding public display environments.

Finally, some middleware limitations are related to security issues; a security framework to protect the data privacy for the users is an interesting topic for future research. On the other hand, the current implementation has been addressed to ad hoc deployments at indoor spaces (e.g., shops or specific places in a mall); large deployments using public displays networks have been addressed by other researches and it may be an interesting extension for the current middleware implementation.

## Figures and Tables

**Table 1:** Message details.

Message		
Field	Type	Description
Uuid	String	Message source identifier
Type	Enum: MessageType	Enumeration with values: Move, Press, Long Press, Tap, Double Tap, Swipe, Drag, Custom, Setup, Ping, Bye, and Feedback
StartContent	String	Payload field for arbitrary data or serialized motion events
EndContent	String	Payload field for arbitrary data or serialized motion events
Extras	String	Payload field for extra data or binary encoded content

**Table 2:** Motion event details.

Motion event		
Field	Type	Description
Id	Integer	Message source identifier
Action	Enum: MessageAction	Enumeration values: Action Down, Action Up, Action Move, Action Cancel, Init, and End
*x*	Integer	*X* coordinate of motion event relative to the screen layout
*y*	Integer	*Y* coordinate of motion event relative to the screen layout
EventTime	Long	Motion event duration in milliseconds
DownTime	Long	First time for user pressed down event, which starts a stream of new events
Source	Integer	Source as specified by the O.S

**Table 3:** Protocol messages details.

Protocol	
Message	Structure
Tap	{
	“uuid”: 49f319b2-9689-44bf-b9fd-a2b076c18c9b,
	“type”: “TAP”,
	“startContent”: {
	“action”: “ACTION_DOWN”,
	“downTime”: 39504973,
	“eventTime”: 39504973,
	“id”: 3,
	“source”: 4098,
	“*x*”: 373.48129999999998,
	“*y*”: 513.59875
	},
	“endContent”: null,
	“extras”: null,
	}

Swipe	{
	“uuid”: 49f319b2-9689-44bf-b9fd-a2b076c18c9b,
	“type”: “DOUBLE_TAP”,
	“startContent”: {
	“action”: “ACTION_DOWN”,
	“downTime”: 40929359,
	“eventTime”: 40929724,
	“id”: 3,
	“source”: 4098,
	“*x*”: 114.84050000000001,
	“*y*”: 1036.1904
	},
	“endContent”: {
	“action”: “ACTION_UP”,
	“downTime”: 40929359,
	“eventTime”: 40929359,
	“id”: 3,
	“source”: 4098,
	“*x*”: 292.59363000000002,
	“*y*”: 646.49492999999995
	},
	“extras”: null,
	}

**Table 4:** Offline tests hardware setup.

Hardware			
	Smartphone	Smart TV	WAMP Broker
Type	Mobile	Google TV	Laptop
CPU	Qualcomm Snapdragon 400. 1.2 GHz	Intel Atom CE4150 1.2 GHz	2.5 GHz
RAM	1 GB	8 GB	8 GB
OS	Android 4.4	Android 3.2	MAC OS X
Network	Wi-Fi 802.11 a/b/n	Wi-Fi 802.11 a/b/n	Ethernet

**Table 5:** Latency and connection time.

System metrics		
	Average (ms)	Standard deviation
Connection time	26.56	7.61
Latency	40.24	9.15
